# A negative survival pressure selection system enables GPCR antagonist screening

**DOI:** 10.1038/s41421-026-00892-7

**Published:** 2026-05-13

**Authors:** Tao Xu, Qinxin Sun, Shuhao Zhang, Tingzhu Liu, Xiaoou Sun, Xusheng Wang, Bin Lin, Zhongjie Liu, Juan Wang, Guodong He, Xiangyu Liu

**Affiliations:** 1 State Key Laboratory of Membrane Biology, Tsinghua-Peking Center for Life Sciences, School of Pharmaceutical Sciences, Tsinghua University, Beijing, China; 2 Beijing Frontier Research Center for Biological Structure, Beijing Advanced Innovation Center for Structural Biology, Tsinghua University, Beijing, China; 3 Memoo (Beijing) Technology Co. Ltd., Beijing, China; 4 School of Basic Medical Sciences, Tsinghua University, Beijing, China; 5 School of Pharmaceutical Sciences (Shenzhen), Sun Yat-Sen University, Shenzhen, Guangdong China; 6 School of Pharmaceutical Sciences (Shenzhen), Shenzhen Campus of Sun Yat-sen University, Sun Yat-sen University, Shenzhen, Guangdong China; 7 Wuya College of Innovation, Shenyang Pharmaceutical University, Shenyang, Liaoning China; 8 Key Laboratory of Structure-Based Drug Design and Discovery of Ministry of Education, Shenyang Pharmaceutical University, Shenyang, Liaoning China; 9 Department of Anesthesiology, Shenzhen Children’s Hospital, Shenzhen, Guangdong China; 10 College of Biotechnology, Tianjin University of Science and Technology, Tianjin, China; 11 Beijing Key Laboratory of Cardiovascular Receptors Research, Peking University, Beijing, China

**Keywords:** Cryoelectron microscopy, Extracellular signalling molecules

Dear Editor,

G protein-coupled receptors (GPCRs) play pivotal roles in regulating physiological processes, and comprehensive statistical analyses indicate that ~36% of approved drugs exert their therapeutic effects by acting on GPCRs, collectively contributing to over 27% of the global pharmaceutical market revenue^1,2^. Over the past several decades, function-based as well as affinity-based GPCR ligand screening methodologies have emerged, including signaling assay-based screening^3^, DNA-encoded library (DEL) screening^4^, yeast surface display (YSD) techniques^5^, and virtual screening^6^. Despite these advances, existing screening methods possess inherent limitations. Affinity-based techniques, such as DEL and YSD, predominantly detect ligand binding events, thereby necessitating subsequent functional validation to comprehensively characterize the activity profiles of the identified ligands. In contrast, functional screening is typically conducted in mammalian cell-based systems, which present a series of operational limitations. For instance, the diversity of GPCR–G protein coupling mechanisms necessitates the customization of detection platforms for individual receptors, which can be expensive or technically demanding^7^. Moreover, mammalian cells are costly to maintain, exhibit sensitivity to specific solvents (e.g., DMSO), and the endogenous expression of GPCRs may potentially interfere with experimental results.

To overcome these challenges, we previously developed a Survival Pressure Selection (SPS) system for GPCR agonist screening^8^. Notably, of all the GPCR-targeting drugs, more than half are antagonists^1^. Here, we present a function-based negative Survival Pressure Selection (nSPS) system for the screening of GPCR antagonists in *Saccharomyces cerevisiae* by employing diphtheria toxin subunit A (DTA)^9^ as the reporter protein and the β_2_-adrenoceptor (β_2_AR) as the model receptor. In this system, DTA is divided into two fragments. The N-terminal domain of DTA (DTA^NTD^, residues 1–131) is inserted into miniGα_s_ between G188 and D189, while the C-terminal domain of DTA (DTA^CTD^, residues 126–191) is fused to the C-terminus of β_2_AR with a 10-amino-acid linker. We term this system the β_2_AR-nSPS system (Supplementary Fig. S1a). Yeast proliferation serves as the primary readout of this assay. In this system, antagonist-mediated disruption of the GPCR–miniG protein interaction prevents reassembly of the toxic DTA, thereby facilitating yeast growth (Fig. 1a). Expression of the construct in yeast resulted in enhanced growth in the presence of the inverse agonist ICI-118551, compared to the ligand-free (Apo) state. In contrast, treatment with 1 µM isoprenaline, a β_2_AR agonist, caused marked growth suppression. Notably, increasing concentrations of ICI-118551 dose-dependently rescued this growth inhibition (Fig. 1b). Similarly, the well-characterized β_2_AR antagonist, carazolol, also alleviated isoprenaline-induced growth suppression (Supplementary Fig. S1b).

**Fig. 1:**
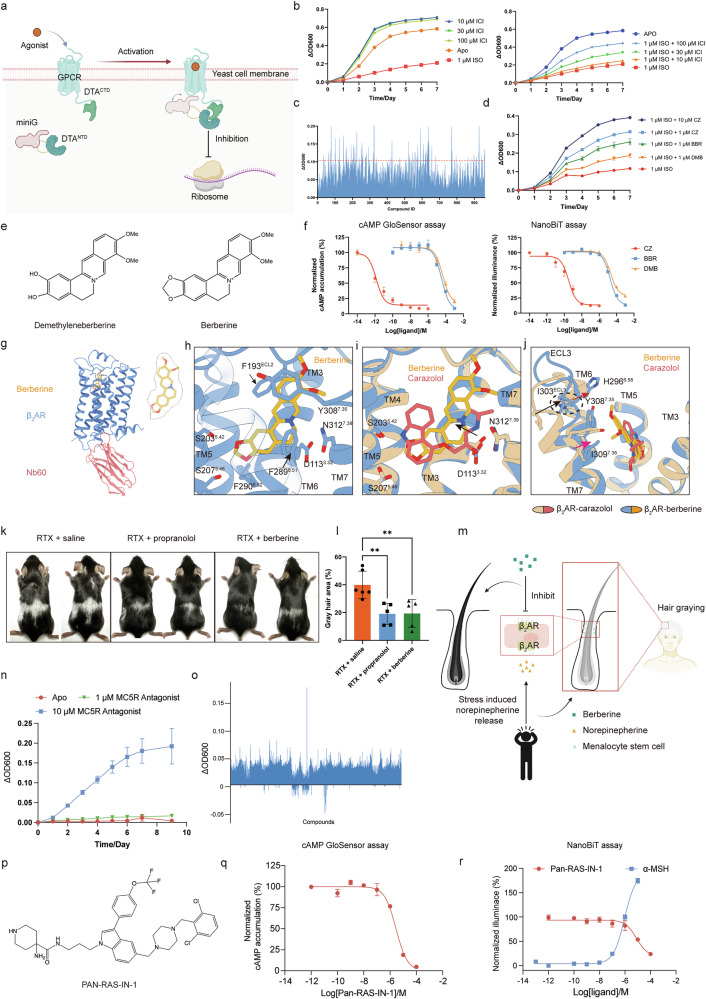
The establishment of the nSPS system and its application to the β_2_AR and MC5R. **a** Schematic diagram showing the nSPS system. Created with BioRender.com. **b** Isoprenaline suppresses β_2_AR-SPS yeast growth rate while ICI-118551 increases the yeast growth rate and rescues the suppression effect of isoprenaline. ISO isoprenaline, ICI ICI-118551. **c** High-throughput screening results for β_2_AR antagonist. Each vertical line represents a compound, and the red dashed line represents the level of increase of 0.105. **d** Carazolol, demethyleneberberine, and berberine rescue the yeast growth suppression effect of isoprenaline. CZ carazolol, DMB demethyleneberberine, BBR berberine. Data are presented as means ± SEM (*n* = 4). **e** Chemical structures of demethyleneberberine and berberine. **f** The GloSensor cAMP and NanoBiT assays confirm demethyleneberberine and berberine as β_2_AR antagonists. Carazolol was employed as a positive control. Data are presented as means ± SEM (*n* = 9). **g** Overall binding mode of berberine in the β_2_AR-T4L–Nb60–berberine complex. **h** Interactions between berberine and the β_2_AR. **i** Detailed comparison of the binding modes of berberine and carazolol at the β_2_AR. **j** Differences in the ECL3 and TM7 of the β_2_AR when bound to berberine and carazolol. **k** Berberine reduces RTX-induced hair graying. *n* = 6 for the RTX only group; *n* = 5 for the RTX + propranolol and RTX + berberine groups. **l** Statistical analysis of the proportions of gray hair area across three groups (**P < 0.005, one-way ANOVA). **m** Illustration of the mechanism by which berberine prevents hair graying. Created with BioRender.com. **n** The MC5R-nSPS yeasts exhibit fast growth in the presence of an MC5R antagonist. Data are represented as means ± SEM (*n* = 4). **o** High-throughput screening for MC5R antagonists. Each vertical line represents a compound. **p** Chemical structure of Pan-RAS-IN-1. **q**, **r** GloSensor cAMP assay (**q**) and NanoBiT assay (**r**) confirm the MC5R antagonist activity of Pan-RAS-IN-1. Data are presented as means ± SEM (*n* = 9).

We successfully established the nSPS method in *S. cerevisiae* using β_2_AR and subsequently assessed its capability for identifying novel GPCR antagonists. As a proof of concept, we implemented a screening pipeline to discover novel antagonists for the β_2_AR from a natural product library consisting of ~1000 compounds (Fig. 1c; Supplementary Fig. S2a). Based on the primary screen results, we selected the top 20 compounds for further functional validation. This process led to the identification of demethyleneberberine as a β_2_AR antagonist. Its structural analog, berberine, was also confirmed as a β_2_AR antagonist. Both compounds reversed isoprenaline-induced growth suppression of the nSPS yeast (Fig. 1d) and exhibited weak antagonistic activity, with the IC_50_ values in the range of 30–40 μM in both the cAMP GloSensor and NanoBiT-based receptor–miniG protein association assays (Fig. 1e, f). Given that berberine exhibited modestly higher potency, we focused our subsequent research on this compound. Additionally, we assessed the selectivity of berberine between the β_2_AR and β_1_AR. The results indicate that berberine also inhibits isoprenaline-induced β_1_AR activation, albeit with slightly lower affinity (Supplementary Fig. S2b).

To further elucidate the structural basis by which berberine antagonizes the β_2_AR signaling, we determined the cryogenic electron microscopy (cryo-EM) structure of the berberine–β_2_AR complex. We utilized a β_2_AR-T4L fusion construct and employed Nb60 to stabilize the receptor’s inactive state^10^ (Supplementary Fig. S3). Following data collection and processing, we resolved the inactive-state structure of the β_2_AR-T4L–Nb60–berberine complex, achieving an overall resolution of 3.04 Å (Fig. 1g; Supplementary Fig. S4 and Table S1; see Materials and Methods section in the Supplementary Information for details). The well-defined electron density map enabled unambiguous modeling of the majority of β_2_AR structure, excluding the T4L portion. The overall structure reveals that β_2_AR adopts an inactive conformation, with berberine occupying the orthosteric binding pocket of β_2_AR (Fig. 1g). Berberine predominantly interacts with β_2_AR through transmembrane helix 3 (TM3), TM5, TM6, TM7 and extracellular loop 2 (ECL2), with its protoberberine moiety oriented vertically in the orthosteric pocket (Fig. 1g). Molecular dynamics (MD) simulations corroborated the stability of this binding pose (Supplementary Fig. S5). The protonated nitrogen atom at the center of the protoberberine moiety forms a conserved polar interaction network with surrounding D113^3.32^ (superscript corresponding to the Ballesteros-Weinstein numbering system^11^) and N312^7.39^. In addition, three aromatic residues in the orthosteric pocket also contribute to the recognition of berberine, including F193^ECL2^, F289^6.51^ (edge-to-surface stacking), and Y308^7.35^ (π–π stacking). On TM5, S203^5.42^ and S207^5.46^ form hydrogen bonds with the dioxolane moiety of berberine (Fig. 1h).

The first high-resolution structure of inactive β_2_ΑR, bound to the antagonist carazolol, was reported in 2007^10^. Structure alignment of berberine-bound and carazolol-bound β_2_ΑR revealed that the receptor adopts nearly identical conformations (the root-mean-square deviation of Cα is 0.5 Å). However, the interaction modes are distinct between berberine and carazolol. Compared to berberine, carazolol mainly occupies the deeper region of the orthosteric pocket, with its carbazole moiety pointing to S203^5.42^ on TM5. On the opposite side, the dimethylamine of carazolol forms polar interactions with D113^3.32^ and N312^7.39^ (Fig. 1h). Notably, the interaction with D113^3.32^ is a hallmark of ligand recognition across many class A GPCRs. Berberine exhibits a greater distance between its protonated nitrogen atom and D113^3.32^ relative to carazolol, which may account for its weaker affinity (Fig. 1i, indicated by the black arrow). Moreover, berberine interacts with the extracellular region of TM7, particularly with Y308^7.35^ (Fig. 1h, j). This additional interaction induces a slight inward shift of the extracellular end of TM7, as reflected by the altered positions of I309^7.36^ and Y308^7.35^ (Fig. 1j). This movement of TM7 is coupled to a coordinated conformational change involving ECL3 (indicated by I303^ECL3^) and a rotation of H296^6.58^, collectively resulting in a rearrangement of the extracellular vestibule compared to the β_2_ΑR–carazolol structure (Fig. 1j).

To validate berberine’s binding mode, we first performed a radioactive ligand competition binding assay, which demonstrated that berberine binds to the orthosteric pocket, as it competes with [^3^H]-dihydroalprenolol ([^3^H]-DHA), like isoprenaline (Supplementary Fig. S6a). To further dissect the interactions, we conducted mutagenesis studies targeting residues that differentially affect berberine and isoprenaline, as mutations in shared key residues abolished the effects of both agonist and antagonist (Supplementary Fig. S6b, c). Berberine uniquely forms π–π stacking interactions with F193^ECL2^ and F289^6.51^, which are not typically observed in isoprenaline binding. F193^ECL2^A mutation disrupts aromatic stacking with berberine’s planar core, while F289^6.51^Y substitution introduces a steric clash due to the added hydroxyl group, selectively impairing berberine binding (Supplementary Fig. S6d–f). Functional assays revealed that both F193^ECL2^A and F289^6.51^Y mutations attenuate berberine-induced rightward shift of the isoprenaline dose–response curve, indicating reduced antagonistic efficacy (Supplementary Fig. S6g). These findings support our structural model, which underlies berberine’s unique binding mode within the orthosteric site.

Emerging research has highlighted the diverse roles of β_2_AR across human tissues and organs, including a critical regulatory function in the hair follicle. Previous studies have indicated that β_2_AR overactivation in melanocyte stem cells (MeSCs) results in MeSC exhaustion and finally leads to hair graying^12^. Several β_2_AR antagonists have demonstrated the ability to reverse hair graying induced by stress. Despite these findings, no drug has been approved currently for the prevention or reversal of hair graying. Berberine, with its long history of clinical use and well-established safety profile in the treatment of metabolic and inflammatory disorders, emerges as a compelling candidate for this indication.

To evaluate its potential for preventing hair graying, we tested berberine’s effect in a stress-induced hair graying mouse model. The transdermal permeation ability of berberine was first assessed to ensure the appropriate dosage during the experiments, and the concentration of 1 mg/mL was used for further study (Supplementary Fig. S7). The stress-induced hair graying model was generated using Resiniferatoxin (RTX) injection. The reported β_2_AR antagonist, propranolol, was used as a positive control. Both propranolol and berberine treatments significantly reduced the average white hair area from ~40% to less than 20% (Fig. 1k, l; Supplementary Fig. S8). These results demonstrate that berberine effectively reverses stress-induced hair graying, exhibiting comparable efficacy to propranolol (Fig. 1m).

Given the conserved interaction patterns observed across all characterized GPCR–G protein complexes to date, the nSPS system holds broad applicability for diverse GPCRs. In practice, the linker length between the receptor and DTA^NTD^ may require optimization for each target. We successfully adopted the nSPS system to G_q_-coupled GPR39 (Supplementary Fig. S9) and G_s_-coupled melanocortin 5 receptor (MC5R)^13^ (Fig. 1n). MC5R plays a key role in governing immune reaction and inflammatory responses. Blocking MC5R with antagonists significantly boosts antitumor immunity and enhances the efficacy of anti-PD-1 immunotherapy, which establishes MC5R as a potential therapeutic target for cancer immunotherapy^13^.

Using this nSPS system, we screened a library of ~4500 compounds against MC5R. Following 7-day monitoring of OD600 under each condition, the top 36 compounds that promoted yeast growth were selected for further characterization (Fig. 1o). Among these candidates, Pan-RAS-IN-1, which is a previously reported pan-Ras inhibitor^14^ (Fig. 1p; Supplementary Fig. S10a), exhibited antagonist-like activity with an IC_50_ of 3 μM in the cAMP GloSensor assay and an IC_50_ of 8 μM in the NanoBiT-based miniG protein association assay (Fig. 1q, r). Analysis of Pan-RAS-IN-1 across four human melanocortin receptor subtypes (MC1R, MC3R, MC4R, MC5R) demonstrated that this compound exhibits weak antagonism at MC1R and MC3R, while displaying moderate activity at MC4R (Supplementary Fig. S10b). Notably, Pan-RAS-IN-1 shows higher affinity for MC5R, with an IC_50_ value more than 10-fold lower than that of MC4R, underscoring its selectivity for MC5R.

In this study, we present a novel method for screening GPCR antagonists by coupling receptor activation to the inhibition of yeast proliferation and GPCR inhibition to yeast growth. A notable feature of the nSPS system is its ability to detect weak antagonists due to the cumulative nature of the yeast growth readout. For example, berberine exhibits an IC_50_ of 30 μM against the β_2_AR, yet it still exhibits robust activity in promoting yeast growth under the screening conditions. This property makes the nSPS method particularly well-suited for primary screening aimed at identifying potential hit compounds. However, given the complexity of the yeast cellular environment, false-positive hits may arise; therefore, rigorous functional validation of primary hits is essential to confirm true antagonist activity.

## Supplementary information

Supplementary Information

## Data Availability

The cryo-EM map and coordinates of β_2_AR–Nb60–berberine have been deposited into the Electron Microscopy Data Bank (EMDB) and Protein Data Bank (PDB) under accession numbers EMD-65599 and 9W3F, respectively.
