# Supplementary material for: A negative survival pressure selection system enables GPCR antagonist screening
Source: Cell Discov. 2026 May 13;12:33. doi: 10.1038/s41421-026-00892-7 (PMC13172303; doi:10.1038/s41421-026-00892-7)
Supplement: Supplementary file 1 — Supplementary Information [file 41421_2026_892_MOESM1_ESM.pdf]

Materials and Methods:

Establishment of nSPS system in yeast

The yeast plasmid transformation was performed using a modified electroporation protocol[1]. Briefly, frozen competent BJ5465 yeast cells were thawed on ice, and 100 μL aliquots were mixed with 1 μg plasmid DNA. The cell-DNA mixture was incubated on ice for 15 min before being transferred to a pre-chilled electroporation cuvette (2 mm, BIO-RAD). Electroporation was conducted using a Gene Pulser Xcell™ system (BIO-RAD) with a single pulse of 500 V for 15 ms. Immediately post-electroporation, cells were resuspended in 2 mL pre-warmed YPD medium (1% yeast extract, 2% peptone, 2% glucose) and incubated at 30°C for 1 h without shaking.

Transformed yeast cells were then pelleted by centrifugation at 2,500 × g for 3 min and resuspended in leucine-deficient culture medium (0.67% yeast nitrogen base, 2% glucose, pH 4.5, 1.6% leucine-deficient dropout supplement). The suspension was plated onto plates containing same selection medium and incubated at 30°C for 48 hr. A single colony was subsequently inoculated into 5 mL selection medium and cultured at 30°C with 250 rpm orbital shaking for 24 hours.

For ligand response assays, yeast cultures were diluted to 1×10^6 cells/mL in induction medium (0.67% yeast nitrogen base, 2% galactose, pH 6.5, 1.6% leucine-deficient dropout supplement) supplemented with different ligand of β_2AR. 50 μL yeast culture were dispensed into 384-well microplates, which were then incubated at 25°C with 85% humidity. The optical density at 600 nm (OD600) was measured for each well at 24-hour intervals. Data analysis was conducted using GraphPad Prism version 10.0.2.

High throughput screening for β_2AR and MC5R antagonists

For the β_2AR, β_2AR-nSPS-BJ5465 yeast was cultivated and diluted as described previously[2]. Different natural products were transferred into 384-well plates (NEST) using an Echo 550 Series Acoustic Liquid Handler (Beckman Coulter) at a volume of 50 nL per well. Subsequently, 50 μl β_2AR-BJ5465-nSPS yeast suspension containing 1 μM isoprenaline (to achieve a basal growth suppression) was added to each well.

For the MC5R, MC5R-nSPS-BJ5465 yeast was cultivated and diluted as described previously. Different compounds were transferred into 384-well plates (NEST) using an Echo 550 Series Acoustic Liquid Handlers (Beckman Coulter) at a volume of 50 nL per well. Subsequently, 50 μl

MC5R-nSPS yeast was added to each well.

The plates were incubated at 25°C, 85% humidity for 7 days. OD600 of each well was monitored every 24 hours using a multiplate reader, and the growth curve of each well was analyzed using MATLAB. The top compounds that promoted yeast growth were selected for further validation.

NanoBiT-based miniG protein recruitment assay

The day before transfection, HEK293T cells were seeded in 6-well plates (NEST) at a density of 2 $\times 10^5$ cells/well and cultured in DMEM (Gibco) supplemented with 10% FBS under 5% CO_2 at 37°C. Transfection was performed when cells reached approximately 80% confluency. For each well, cells were co-transfected with a mixture of receptor-SmBiT and LgBiT-miniGαs using polyethylenimine (PEI) as transfection reagent. Cells were harvested 24 h post-transfection and resuspended in HBSS containing 20 mM HEPES (pH 7.5) and 10 μM coelenterazine and aliquoted into white 96-well plates (NEST) at a volume of 90 μl.

For the β_2AR, to assess the antagonist activities of berberine and demethyleneberberine, cells were equilibrated at room temperature for 1 hour, after which varying concentrations of each compound were added to individual wells, while carazolol served as a positive control. Following a 10-minute incubation, 10 nM isoprenaline was added to stimulate miniGαs recruitment. Luminescence of each well was measured at 1-minute intervals. Dose–response curves were generated by nonlinear regression analysis using GraphPad Prism 10.

For the MC5R, varying concentrations of Pan-RAS-IN-1 were added to individual wells. Following a 10-minute incubation, 10 nM α-melanocyte-stimulating hormone (α-MSH) was added to stimulate miniGαs recruitment. Luminescence was measured at 1-minute intervals in each well. Dose–response curves were generated by nonlinear regression analysis using GraphPad Prism 10.0

cAMP GloSensor assay

β_2AR and MC5R activation leads to cAMP accumulation through Gαs, which can be detected through the GloSensor™ reporter system. CHO cells were utilized to minimize the interference from endogenous β_2AR. The day before transfection, CHO cells were seeded in 6-well plates at the density of 2 × 10^5 cells/well and cultured in DMEM/F12 (Gibco) supplemented with 10% FBS

under 5% CO_2 at 37°C. After overnight growth, CHO cells were co-transfected with 100 ng β_2AR and 2.5 μg pGloSensor™-22F cAMP plasmid (Promega) using PEI as transfection reagent. For MC5R, HEK293T cells were seeded in 6-well plates (NEST) at a density of 2×10^5 cells/well and cultured in DMEM (Gibco) supplemented with 10% FBS under 5% CO_2 at 37°C at the day before transfection. For each well, cells were co-transfected with 1.25 μg MC5R and 3.75 μg pGloSensor™-22F cAMP plasmid using PEI as transfection reagent.

After 24 h culture, cells were harvested and resuspended in HBSS buffer containing 20 mM HEPES (pH 7.5) and 150 μg/mL D-luciferin. The suspension was aliquoted into white 96-well plates at a volume of 90 μl. After equilibration at 37°C for 1 hour and subsequent incubation for 1 hour at room temperature, various concentrations of berberine were added to each well and baseline luminescence was measured after 10 minutes. Then, 100 nM isoprenaline was added to each well to stimulate β_2AR activation. Real-time luminescence was recorded at 1 min intervals for 15 min. Dose-response curves were generated using nonlinear regression analysis in GraphPad Prism 10. For the MC5R, after cells were aliquoted and equilibrated, various concentrations of Pan-RAS-IN-1 were added to each well and baseline luminescence was measured after 10 minutes. Then, 10 nM α-MSH was added to each well to stimulate MC5R activation. Real-time luminescence was recorded at 1 min intervals for 15 min. Dose-response curves were generated using nonlinear regression analysis in GraphPad Prism 10.0

β_2AR-T_4L-Nb60 complex expression and purification

Nb60 was expressed and purified as previously reported[2]. In brief, E. *coli BL21(DE3)* cells transformed with Nb60-6×His were cultured in Terrific Broth (TB) medium at 37°C with 220 rpm shaking. When the OD600 reached 1.0 (approximately 4 hours), protein expression was induced by the addition of 1 mM IPTG, followed by overnight incubation at 20°C.

After overnight expression, cell pellets were resuspended in periplasmic extraction buffer (30 mM Tris-HCl pH 8.0, 20% sucrose) containing 1 mg/mL lysozyme and protease inhibitor. The suspension was incubated on ice for 45 minutes and the periplasmic Nb60 was obtained by osmotic shock. After centrifugation, the supernatant containing Nb60 was purified by Ni-NTA chromatography. Further purification was achieved through size-exclusion chromatography (SEC) on Superdex 75 10/300 GL column (Cytiva).

The β_2AR-T4L was expressed and purified as previously reported[2]. In brief, the β_2AR-T4L was expressed in *Sf*9 insect cells using recombinant baculovirus (Bac-to-Bac expression system) for 48 h at 27 °C. After expression, cell pellets were thawed in lysis buffer (20 mM Tris-HCl pH 7.5, 1 mM EDTA) supplemented with 20 μg/mL leupeptin (Sigma), 160 μg/mL benzamidine (Sigma), and 2 mg/mL iodoacetamide (Sigma). Cell membranes were collected by centrifugation at 18,000 rpm for 45 min at 4°C and homogenized using a glass Dounce homogenizer.

The membranes were then solubilized in 20 mM HEPES pH 7.5, 100 mM NaCl, 10 μM tris(2-carboxyethyl)phosphine (TCEP) containing 1% n-dodecyl-β-D-maltopyranoside (DDM, Anatrace) and 0.1% cholesterol hemisuccinate (CHS, Sigma) for 1.5 hr at 4°C. Nb60 was added at 10 μg/mL during solubilization to stabilize the inactive state of β_2AR. The solubilized protein was loaded onto M1 anti-FLAG affinity resin, and the detergent was exchanged to 0.01% lauryl maltose neopentyl glycol (LMNG, Anatrace). After removal of unwanted contaminations, the complex was eluted with 0.2 mg/mL FLAG peptide in 20 mM HEPES pH 7.5, 100 mM NaCl, 0.01% LMNG, 0.001% CHS, 10 μM TCEP and 5 mM EDTA. The eluate was then concentrated using 50 kDa cutoff centrifugal filter (Millipore) and incubated with a 1.5 molar excess of Nb60 at 4°C for 1 hour. Further purification was performed by SEC using Superdex 200 Increase column (Cytiva) equilibrated with 20 mM HEPES pH 7.5, 100 mM NaCl, 0.002% LMNG, 0.0002% CHS. The monodisperse peak fractions corresponding to the β_2AR-T4L-Nb60 complex were pooled and concentrated to about 7 mg/ml for cryo-EM grid preparation.

Cryo-EM sample preparation and data collection

Prior to grid preparation, the β_2AR-T4L-Nb60 complex was incubated with 1 mM berberine (TargetMol) on ice for 2 hours. After centrifugation, 4 μL protein complex was applied onto the glow-discharged cryo-EM grids (Quantifoil Au R1.2/1.3 300 mesh), then the blotted grid (4 s, 8 ℃, 100% humidity) was rapidly plunged into liquid ethane cooled by liquid nitrogen using a Vitrobot (Thermo Fisher Scientific).

Cryo-EM data were collected on a 300 kV Titan Krios transmission electron microscope (Thermo Fisher Scientific). Automated data collection was performed using EPU v3.0 (Thermo Fisher Scientific) with a Falcon4 electron detector. Micrographs were acquired at a pixel size of 0.808 Å with a defocus range of -1.1 to -1.6 μm. The total dose was about 50 $e^{-}/Å^{2}$ for each stack,

and all 32 frames were aligned and summed using MotionCor2[3].

Cryo-EM data processing

Initial image processing was performed using cryoSPARC (Structura Biotechnology Inc). A total of 3,415 micrographs were collected from which 3,627,752 particles were automatically picked using the blob picker and extracted. Three rounds of 2D classification were performed, and particles with clear β_2AR–Nb60 features were manually selected for downstream reconstruction.

Ab initio reconstruction was employed to generate three initial maps. Subsequent heterogeneous refinements were utilized to progressively remove poorly aligned particles. After three rounds of heterogeneous refinements, 705,731 particles exhibiting defined secondary structures were retained and were re-extracted (box size=228) for further refinement. After heterogeneous refinements, non-uniform refinements and local refinements, a final set of 347,166 particles were refined to a global resolution of 3.04 Å. The local resolution was estimated in cryoSPARC using default parameters.

Model building and refinement

The β_2AR-T4L structure (PDB ID: 2RH1) and Nb60 structure (PDB ID: 5JQH) were used as initial models, which were docked into the cryo-EM density map using UCSF ChimeraX[4]. The restraint file for berberine was generated using PHENIX eLBOW. The model was manually adjusted in COOT[5] and iteratively refined using PHENIX. The final model was validated using PHENIX[6]. All structural figures were prepared using UCSF ChimeraX.

MD simulation

All simulations used the CHARMM36m parameter set (with CMAP protein backbone energy correction terms) for proteins, lipids and salt ions and the CHARMM TIP3P water model. Force field parameters for berberine were generated using the ParamChem server. Penalties were checked to ensure they were suitable for MD simulations.

All simulations were performed on a single graphics processing unit using Amber22 Compute Unified Device Architecture (CUDA) version of particle-mesh Ewald MD. Prepared protein structures were inserted into a large equilibrated POPC (1-palmitoyl-2-oleoyl-sn-glycero-3-

phosphocholine) bilayer solvated with 0.15 M NaCl. The total number of atoms or the approximate system size is ~85,000 atoms. Heating (to 310 K over 150 ps) and equilibration (50 ns with restraints on protein and ligand) steps were performed before production simulations. Production simulations were performed in the NPT ensemble at 310 K and 1 bar, using a Langevin thermostat for temperature coupling and a Monte Carlo barostat for pressure coupling. Bond lengths to hydrogen atoms were constrained using SHAKE. Non-bonded interactions were cut off at 12 Å. Trajectory snapshots were saved every 200 ps. All simulations were 1000 ns in length. Three independent simulations were performed for each complex. The CPPTRAJ package in AmberTools23 was used for post-analysis. Visual Molecular Dynamics (VMD) was used to visualize the trajectories. Time traces from the simulations were smoothed using a moving average with a window size of 10 ns.

Radioactive ligand competition binding assay

The β_2AR-T4L protein was expressed and harvested as previously described (see method). For membrane preparation, cell pellets obtained from 200 mL of culture were lysed and homogenized in 40 mL of lysis buffer (20 mM HEPES, pH 7.5, 2 mM EDTA). The lysate was first centrifuged at 800 rpm for 20 min to remove unbroken cells and debris. The resulting supernatant was then ultracentrifuged at 13,000 rpm for 20 min to pellet the membrane fraction. The membrane pellet was resuspended in binding buffer (20 mM HEPES, pH 7.5, 100 mM NaCl).

For radioligand binding assays, 25 μL of the fresh prepared membrane suspension was incubated with varying concentrations of berberine and isoprenaline in the presence of 1 nM $[^3H]$-DHA (dihydroalprenolol hydroxychloride). The reaction volume was adjusted to 250 μL with binding buffer and incubated at room temperature for 1.5 h with gentle shaking (80 rpm). Following incubation, membranes were collected by vacuum filtration through Whatman GF/B glass fiber filters and washed three times with ice-cold binding buffer to remove unbound hot ligand. Filters containing bound membranes were transferred to scintillation vials, immersed in 2.5 mL of OptiPhase HiSafe 3 scintillation cocktail, and allowed to extract overnight in the dark. Radioactivity was quantified using a MicroBeta2 scintillation counter.

Specific binding was determined by subtracting nonspecific binding (measured in the presence of excess unlabeled ligand) from total binding. Binding curves were fitted using nonlinear regression analysis in GraphPad Prism 10.0.

Mouse transdermal permeation experiment

All animal experimental procedures were conducted in accordance with internationally accepted principles for laboratory animal use and care, as outlined in the European Community guidelines (EEC Directive of 1986; 86/609/EEC). The study was approved by the Institutional Animal Care and Use Committee of Sun Yat-sen University (Approval No. SYSU-YXYSZ-20210332). The experiment was performed using vertical Franz-type diffusion cells. The supply liquid contained 4 mL of test compounds: 1 mg/mL or 10 mg/mL of propranolol (positive control) or berberine dissolved in a mixture of absolute ethanol: 1,2-propanediol: menthone (57:38:5, v/v/v). The acceptance liquid consists of 50 mL 1% phosphate-buffered saline (PBS, pH 7.4) (w/v). The full-thickness dorsal skin from mice (4 cm × 4 cm) was mounted between the supply liquid and the acceptance liquid with stratum corneum facing the supply liquid. 1 mL of acceptance solution was measured at 0.25, 0.5, 1, 1.5, 2, 3, 4, 6, and 7 h post-application. The UV absorbance at wavelength of 292 nm (propranolol), 231 and 266 nm (berberine) were measured, and the UV absorbance-time curve was plotted using GraphPad prism 10.

Mouse stress-induced hair graying rescue experiment

RTX was dissolved in phosphate-buffered saline (PBS, pH 7.4) containing 2% (v/v) dimethyl sulfoxide and 0.15% (v/v) Tween 80, with a stock concentration of 5 μg/mL. Propranolol and berberine were dissolved in solution consisting of ethane: 1,2-Propanediol: peppermint ketone = 57: 38: 5. Sixteen 4–6-week-old female C57BL/6 mice were randomly allocated into three experimental groups: RTX + saline (n=6), RTX + propranolol (n=5), and RTX + berberine (n=5). Following dorsal hair removal using electric clippers, 400 μL of test compound was topically applied to the exposed skin. 1-hour post-application, RTX (20 μg/kg) was administered via triple lateral abdominal injections. Subsequent compound application was performed 11 hr post-RTX injection. Photographic documentation and quantitative analysis of cutaneous responses were conducted on day 24 post-treatment initiation.

Reference：

1 McMahon, C. *et al.* Yeast surface display platform for rapid discovery of conformationally selective nanobodies. *Nature Structural & Molecular Biology* **25**, 289-296, doi:10.1038/s41594-018-0028-6 (2018).

2 Staus, D. P. *et al.* Allosteric nanobodies reveal the dynamic range and diverse mechanisms of G-protein-coupled receptor activation. *Nature* **535**, 448-452, doi:10.1038/nature18636 (2016).

3 Zheng, S. Q. *et al.* MotionCor2: anisotropic correction of beam-induced motion for improved cryo-electron microscopy. *Nature methods* **14**, 331-332, doi:10.1038/nmeth.4193 (2017).

4 Pettersen, E. F. *et al.* UCSF ChimeraX: Structure visualization for researchers, educators, and developers. *Protein Sci* **30**, 70-82, doi:10.1002/pro.3943 (2021).

5 Emsley, P. & Cowtan, K. Coot: model-building tools for molecular graphics. *Acta crystallographica. Section D, Biological crystallography* **60**, 2126-2132, doi:10.1107/s0907444904019158 (2004).

6 Adams, P. D. *et al.* PHENIX: a comprehensive Python-based system for macromolecular structure solution. *Acta crystallographica. Section D, Biological crystallography* **66**, 213-221, doi:10.1107/s0907444909052925 (2010).

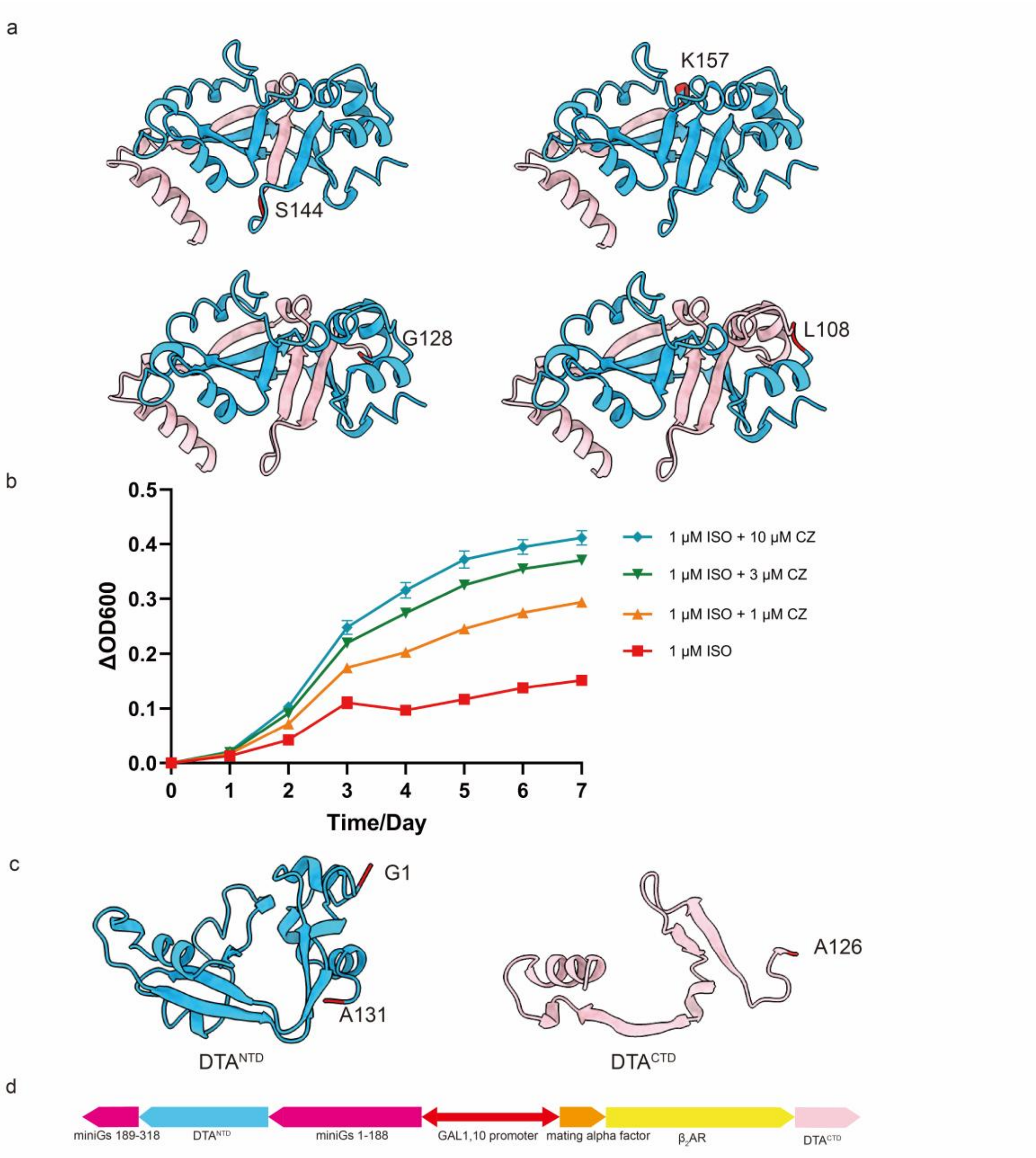

Supplementary information, Fig. 1| Establishment of nSPS system.

a. Four different strategies for splitting DTA. DTA was split into the N-terminal domain (NTD; cyan) and C-terminal domain (CTD; pink). The split point is colored red, and the split residue is labeled.

b. Carazolol (CZ) rescues suppression effect of isoprenaline (ISO). Data are represented as mean ± SEM (n=4).

c. The split strategy that involves NTD (1-131) and CTD (126-191) results in the successful establishment of the nSPS system.

d. The gene architecture of the nSPS system.

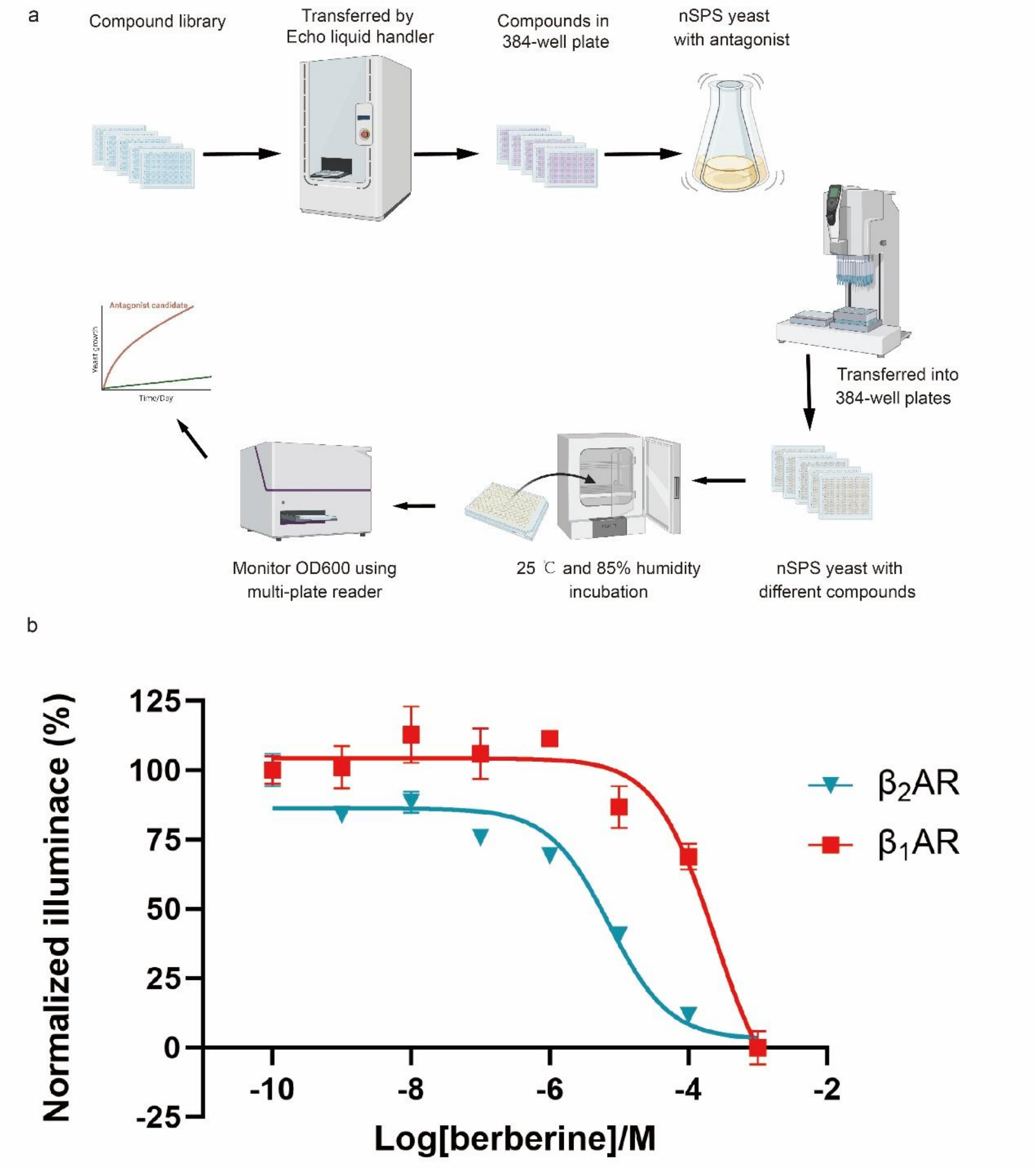

Supplementary information, Fig. 2| The identification of berberine as a β$_2$AR antagonist using nSPS method.

a. The overall workflow of the high throughput screening using nSPS system. Created with BioRender.com.

b. The selectivity of berberine between the β$_2$AR and the β$_1$AR. Data are represented as mean ± SEM (n=3).

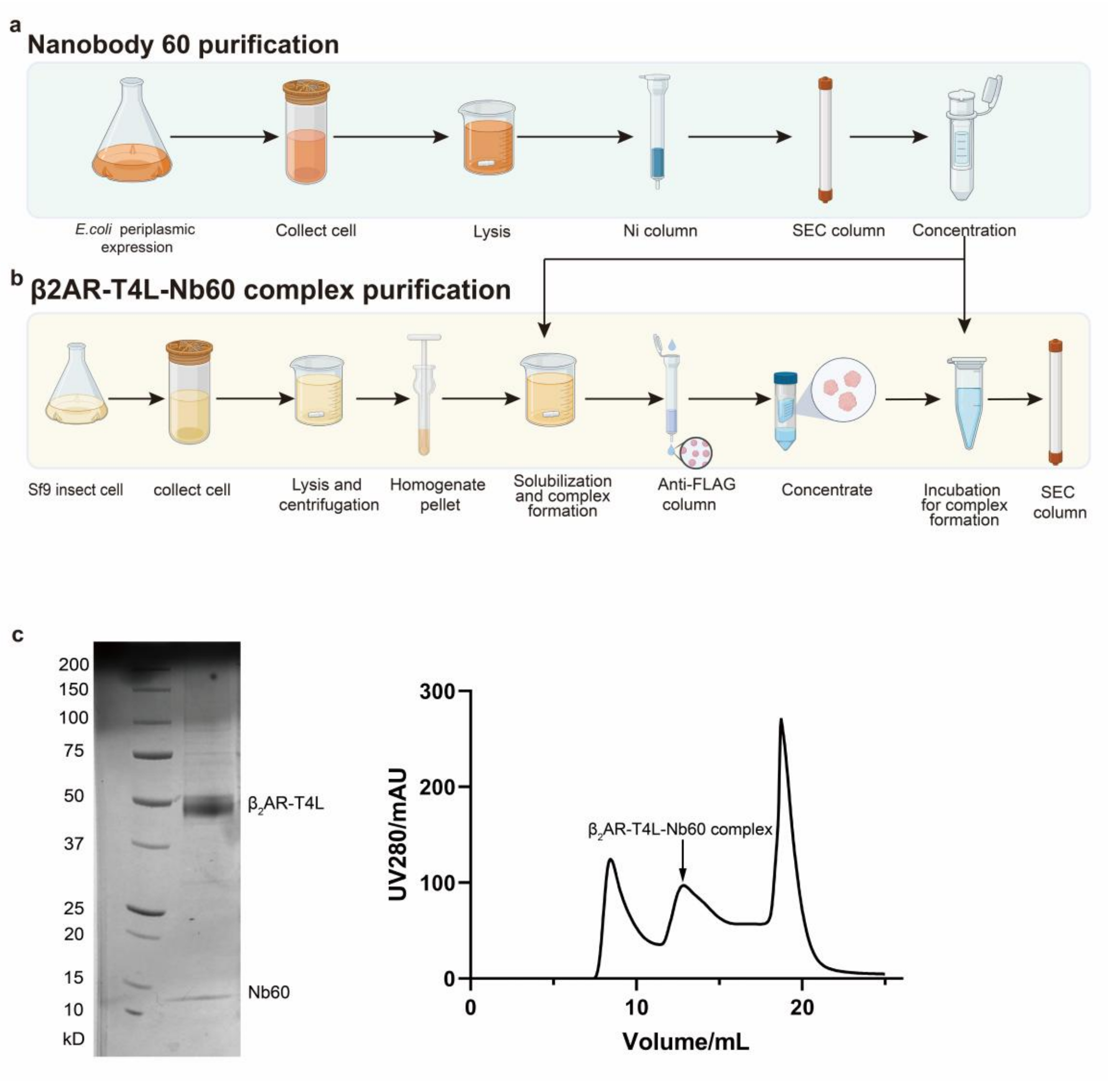

Supplementary information, Fig. 3| The purification of the β_2AR-T4L-Nb60 complex.

a. The protein expression and purification procedures of Nb60. Created with BioRender.com.

b. The purification procedures of the β_2AR-T4L-Nb60 complex. Created with BioRender.com.

c. The SDS-PAGE and size exclusion chromatography (SEC) results of protein purification.

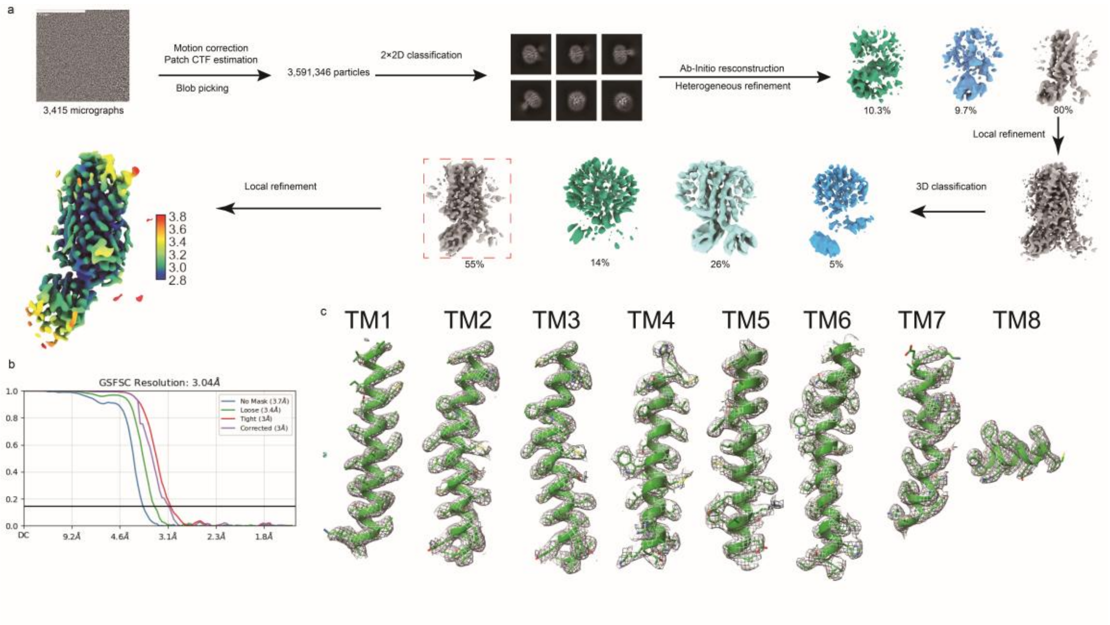

Supplementary information, Fig. 4| Cryo-EM data processing workflow the β$_2$AR-T4L-Nb60 complex.

a. The Data processing workflow of β$_2$AR-T4L-Nb60 complex All movies were motion-corrected using MotionCor2 and then imported into cryoSPARC (V.4.5). Final density map was colored according to the local resolution (Å) of the map.

b. The Gold-standard Fourier Shell Correlation (GSFSC) curve indicates that the resolution of the final cryo-EM map of the β$_2$AR-T4L-Nb60 complex is 3.04 Å.

c. Model and Cryo-EM densities of transmembrane helices of β$_2$AR

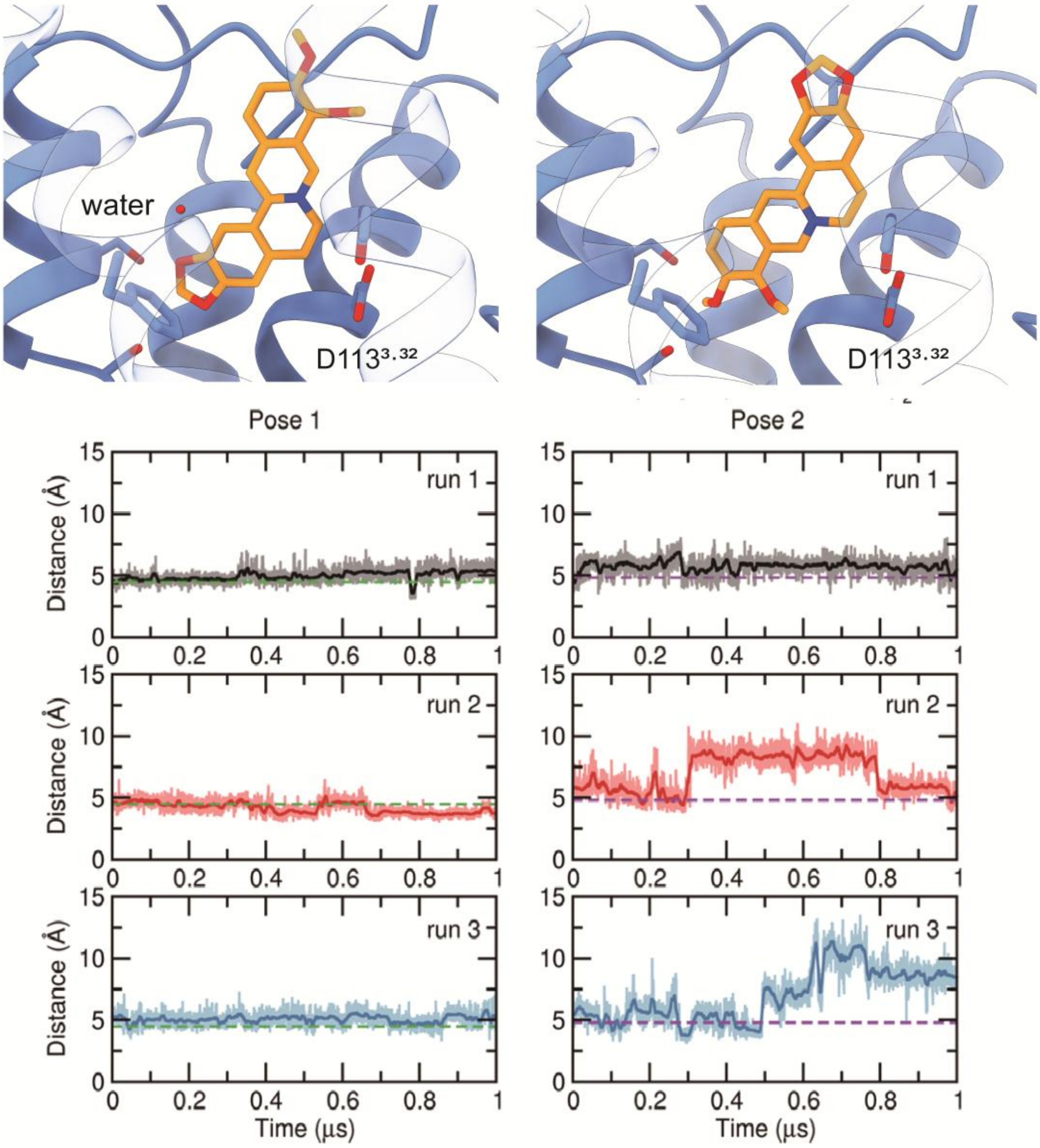

Supplementary information, Fig. 5| Distance between the nitrogen atom in berberine and the carboxyl group in D113$^{3.32}$ of β$_2$AR.

Time evolution of the distance between the quaternary nitrogen atom of berberine and the carboxylate group of D113$^{3.32}$ in the β$_2$AR during MD simulations. Distances are shown for three independent 1-μs replicates for each binding orientation (Pose 1, left; Pose 2, right). Light traces represent raw distances, and bold lines correspond to 10-ns running averages. Dashed lines indicate average N–O (carboxylate) distances from the cryo-EM structures (Pose 1: 4.5 Å; Pose 2: 4.8 Å).

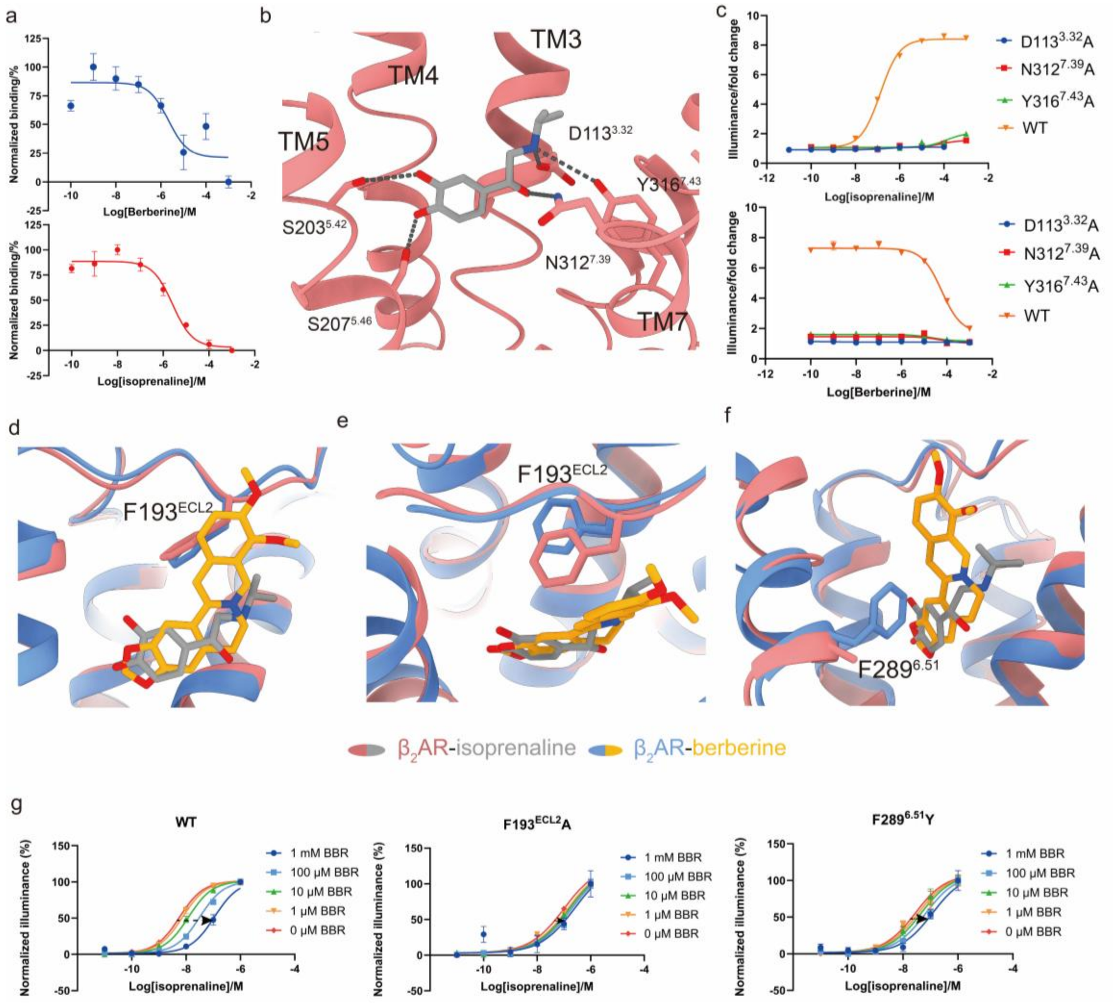

Supplementary information, Fig. 6|. Mutagenesis validation of berberine's binding mode in β_2AR.

a. Radioactive ligand competition binding of berberine and isoprenaline with [3H]-DHA. Data are represented as mean ± SEM (n=4).

b. Orthosteric binding pocket of the β_2AR–isoprenaline complex (PDB ID: 7DHR).

c. Mutations at key residues disrupt both agonist (isoprenaline) and antagonist (berberine) effects due to shared binding site.

d,e. F193^{ECL2} adopts distinct conformations in the berberine- and isoprenaline-bound structures, with π–π stacking observed only with berberine.

f. The side chain of F289$^{6.51}$ orients toward the center of berberine, forming an additional π–π interaction which is absent upon isoprenaline binding.

g. Concentration-response curves of isoprenaline-induced β_2AR activation in wild-type, F193^{ECL2}A, and F289$^{6.51}$Y mutants in the presence of increasing berberine concentrations (0, 1 μM, 10 μM, 100 μM, 1 mM). pA$_2$ values from Schild analysis were 5.095 (WT), 3.808 (F193^{ECL2}A), and 4.441 (F289$^{6.51}$Y). Data are presented as mean ± SEM (N=6).

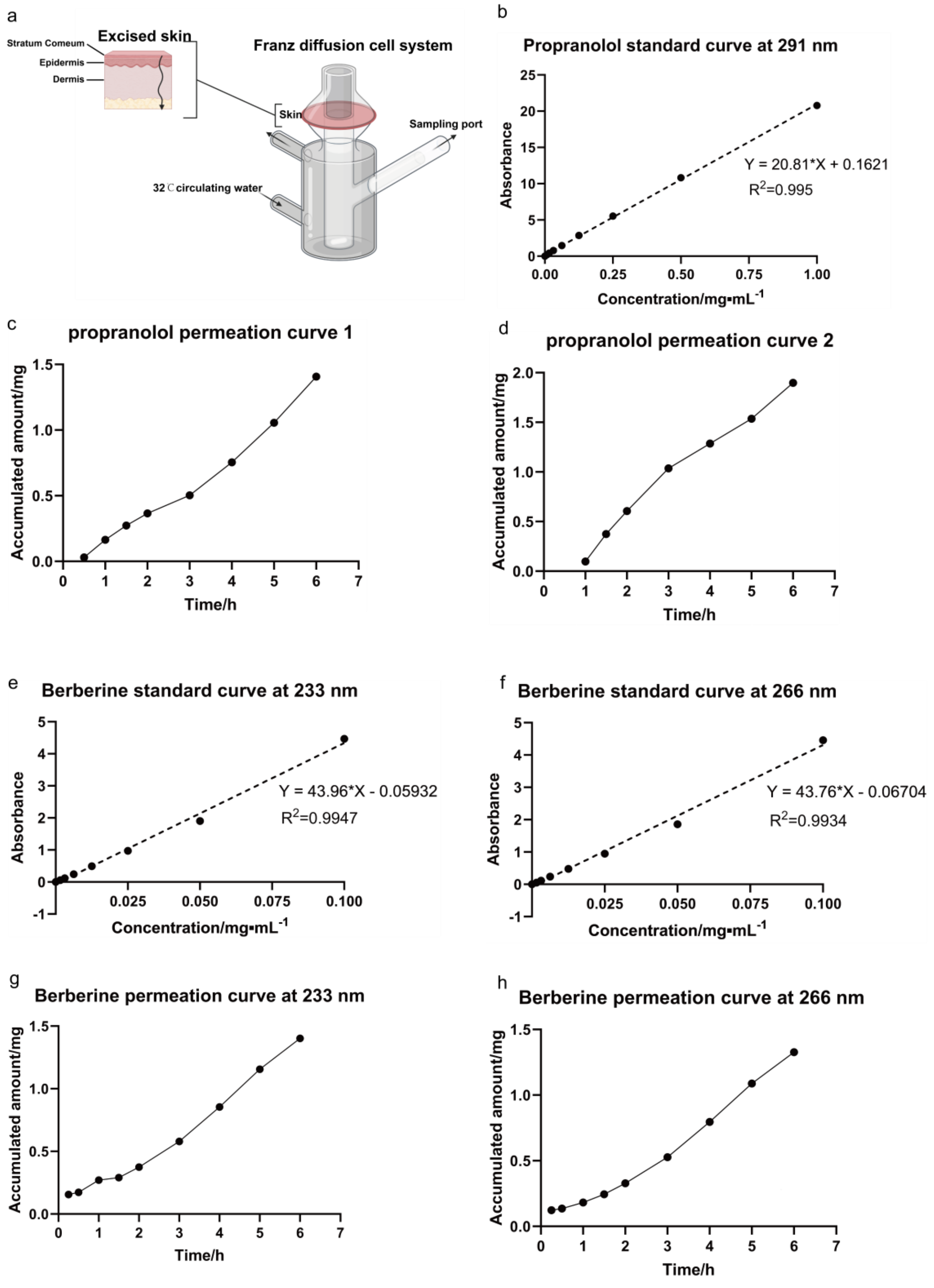

Supplementary information, Fig. 7| Propranolol/Berberine transdermal permeation experiment.

a. The illustration of experiment device. Created with BioRender.com

b. The transdermal permeation experiments results of propranolol standard at 291 nm**.**,

c,d. Propranolol permeation curves.

e. Berberine standard curve at 233 nm.

f. Berberine standard curve at 266 nm.

g. Berberine permeation curve at 233 nm.
h. Berberine permeation curve at 266 nm.

a

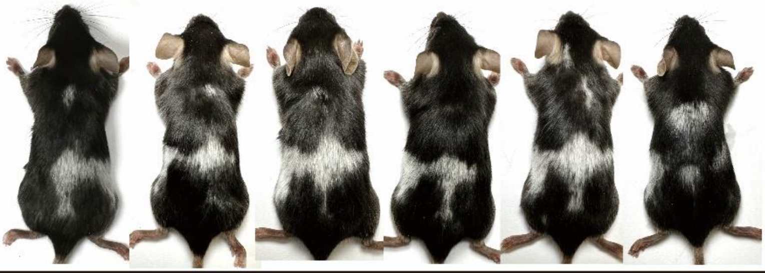

Mouse No.	1	2	3	4	5	6
Total back area (μm^2)	7.6×10^6	1.3×10^7	1.8×10^7	6.4×10^6	1.3×10^7	7.7×10^6
White hair area (μm^2)	2.6×10^6	3.8×10^6	9.8×10^6	2.3×10^6	6.4×10^6	2.8×10^6
Proportion (%)	34.66	29.43	53.84	34.70	50.59	36.15

b

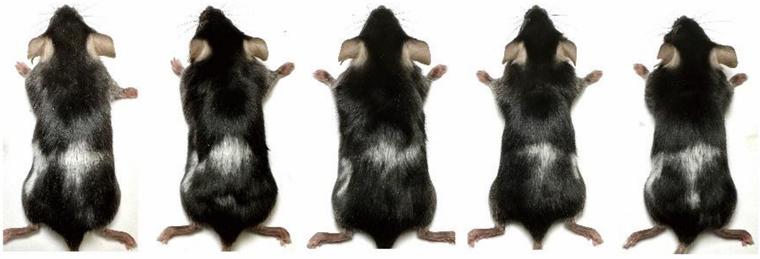

Mouse No.	1	2	3	4	5
Total back area (μm^2)	7.1×10^6	6.2×10^6	4.4×10^6	5.0×10^6	6.6×10^6
White hair area (μm^2)	1.9×10^6	1.6×10^5	4.8×10^5	5.5×10^5	1.4×10^6
Proportion (%)	26.65	25.73	10.88	11.13	20.88

c

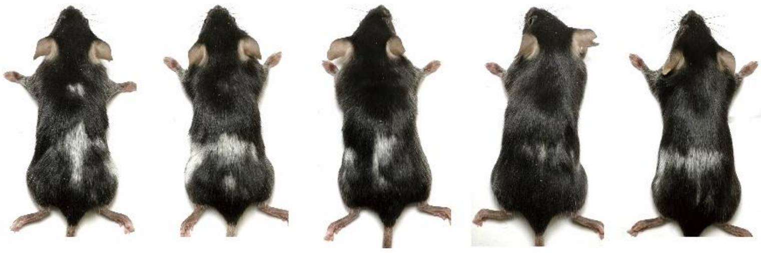

Mouse No.	1	2	3	4	5
Total back area (μm^2)	6.8×10^6	1.2×10^7	5.0×10^6	6.5×10^6	6.7×10^6
White hair area (μm^2)	1.9×10^6	3.1×10^6	1.3×10^6	4.2×10^5	7.1×10^5
Proportion (%)	28.39	26.34	24.93	6.46	10.62

Supplementary information, Fig. 8| White-hair prevention effect of different compounds. RTX induces hair greying of C57/BL6 mice with different compounds: **a.** saline, **b.** propranolol, **c.** Berberine.

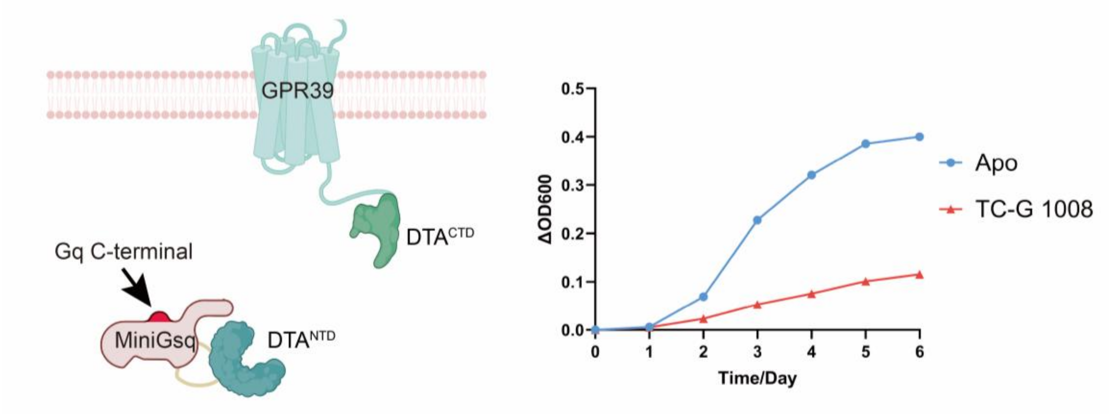

Supplementary information, Fig. 9| nSPS establishment using GPR39 and miniGsq.

TC-G 1008, a reported agonist of GPR39, suppresses GPR39-nSPS yeast growth. Data are presented as mean ± SEM (n=4).

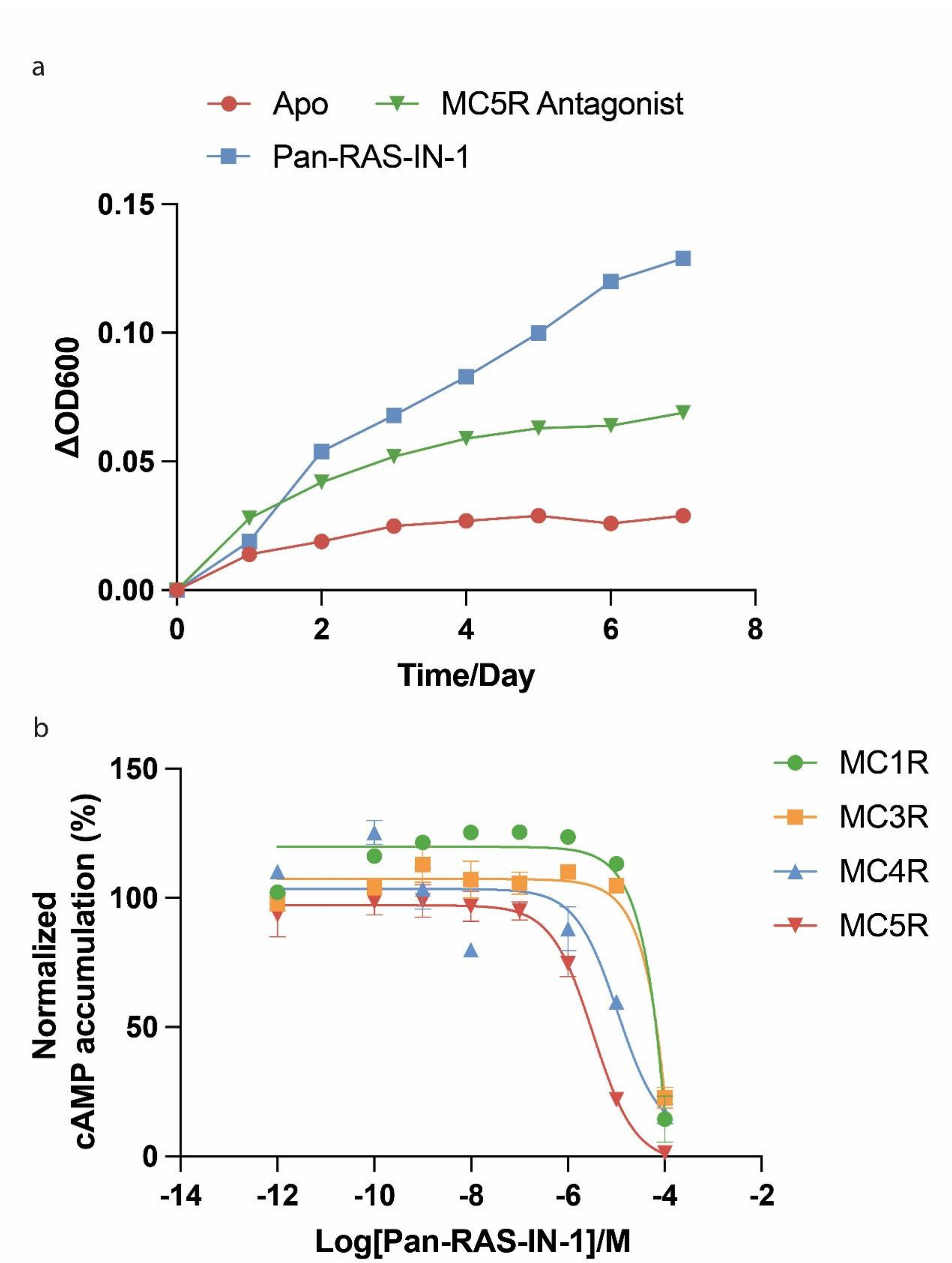

Supplementary information, Fig. 10| Pan-RAS-IN-1 increases MC5R-nSPS yeast growth rate and exhibits MC5R selectivity.

a. Pan-RAS-IN-1 increases MC5R-nSPS yeast growth rate.

b. Pan-RAS-IN-1 exhibits MC5R selectivity over MC1R, MC3R and MC4R as measured by cAMP Glo-sensor assay. Data are represented as mean ± SEM (n=3). Y axis values are normalized to the maximum response of α-MSH for each melanocortin receptor subtype.

Supplementary information, Table S1. | Cryo-EM data collection, refinement and validation statistics

	β_2AR-T4L-Nb60-Berberine (PDB: 9W3F) (EMDB: EMD-65599)
**Data collection and processing**	
Magnification	96000
Voltage (kV)	300
Electron exposure (e–/Å^2)	50
Defocus range (μm)	-1.1~-1.6
Pixel size (Å)	0.808
Symmetry imposed	C1
Initial particle images (no.)	3,591,346
Final particle images (no.)	347166
Map resolution (Å)	3.04
FSC threshold	0.143
Map resolution range (Å)	2.75-12.32
**Refinement**	
Initial model used (PDB code)	2RH1 and 5JQH
Model resolution (Å)	3.41
FSC threshold	0.5
Model resolution range (Å)	2.97-3.41
Map sharpening *B* factor (Å^2)	183.5
Model composition	
Non-hydrogen atoms	2995
Protein residues	393
Ligands	1
B factors (Å^2)	
Protein	91.4
Ligand	83.43
R.m.s. deviations	
Bond lengths (Å)	0.003
Bond angles (°)	0.458
Validation	
MolProbity score	1.36
Clashscore	4.23
Poor rotamers (%)	0
Ramachandran plot	
Favored (%)	97.16
Allowed (%)	2.84
Disallowed (%)	0.00
